# *Citrus sinensis* Annotation Project (CAP): A Comprehensive Database for Sweet Orange Genome

**DOI:** 10.1371/journal.pone.0087723

**Published:** 2014-01-28

**Authors:** Jia Wang, Dijun Chen, Yang Lei, Ji-Wei Chang, Bao-Hai Hao, Feng Xing, Sen Li, Qiang Xu, Xiu-Xin Deng, Ling-Ling Chen

**Affiliations:** 1 Center for Bioinformatics, College of Life Science and Technology, Huazhong Agricultural University, Wuhan, P.R. China; 2 School of Science, Huazhong Agricultural University, Wuhan, P.R. China; 3 Key Laboratory of Horticultural Plant Biology of Ministry of Education, Huazhong Agricultural University, Wuhan, P.R. China; Wuhan University, China

## Abstract

*Citrus* is one of the most important and widely grown fruit crop with global production ranking firstly among all the fruit crops in the world. Sweet orange accounts for more than half of the *Citrus* production both in fresh fruit and processed juice. We have sequenced the draft genome of a double-haploid sweet orange (*C. sinensis* cv. Valencia), and constructed the *Citrus sinensis* annotation project (CAP) to store and visualize the sequenced genomic and transcriptome data. CAP provides GBrowse-based organization of sweet orange genomic data, which integrates *ab initio* gene prediction, EST, RNA-seq and RNA-paired end tag (RNA-PET) evidence-based gene annotation. Furthermore, we provide a user-friendly web interface to show the predicted protein-protein interactions (PPIs) and metabolic pathways in sweet orange. CAP provides comprehensive information beneficial to the researchers of sweet orange and other woody plants, which is freely available at http://citrus.hzau.edu.cn/.

## Introduction

*Citrus* is one of the most important and widely grown fruit crop in the world, with global production and total acreage ranking firstly among all the fruit crops. *Citrus* is a large genus with more than ten major species. Among them, sweet orange is responsible for about 60% of production for both fresh fruit and processed juice consumption [1]. Besides their economical and nutritional importance, *Citrus* fruits also have unique botanical characteristics such as nucellar embryony [2]. Normal sweet oranges are diploids with nine pair of chromosomes, and the estimated genome size is about 367 Mb [2]. Recently, we sequenced the draft genome of a double-haploid sweet orange (*C. sinensis* cv. Valencia) by using whole genome shotgun approach combined with long paired-end DNA sequencing technology [3]. The double-haploid genome was assembled to 4,811 scaffolds with N50 equal to 1.7 Mb. The total contig length (320.5 Mb) covers about 87% of the sweet orange genome, and scaffolds were aligned and oriented to the *Citrus* linkage map, about 80% of the assembled genome was anchored to nine pseudo-chromosomes [3]. An integrative strategy combining *ab initio* gene prediction, homology search, and experimental evidence including expressed sequence tags (ESTs), RNA-seq and RNA-paired end tags (RNA-PETs) was employed to annotate protein-coding genes in sweet orange genome, finally we obtained 29,445 protein-coding gene loci with 44,387 transcripts [3]. The availability of the sweet orange genome sequence provides a valuable genomic resource for citrus genetics and breeding improvement. To intuitively provide the sweet orange genome sequence and annotation, we constructed *Citrus sinensis* annotation project (CAP), which is a portal site for various types of sweet orange data.

CAP provides an integrative platform for GBrowse-based organization of sweet orange genomic data and links many public databases, which includes overview of the pseudo-chromosomes and scaffolds, gene annotation containing *ab initio* gene prediction, EST, RNA-seq and RNA-PET evidence. Detailed protein coding gene information is provided in a keyword search system including predicted function, homologs in model plants, RNA and protein fold prediction and transcriptome evidence. In addition, we construct a user-friendly web interface to show the predicted protein-protein interactions (PPIs) in sweet orange, and supply metabolic pathways based on the Kyoto Encyclopedia of Genes and Genomes (KEGG) pathways [4]. CAP can provide comprehensive information beneficial to the researchers of sweet orange and other woody plants.

## Results and Discussion

### Gene annotation

**Figure 1:**
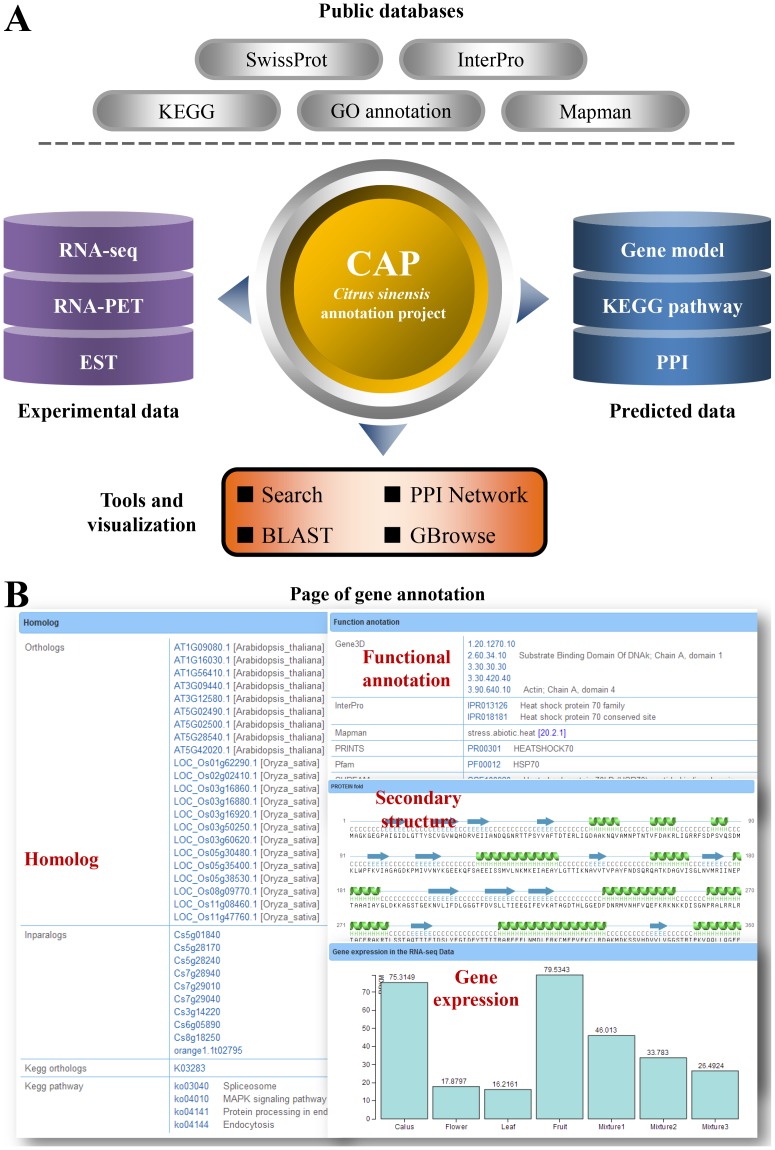
The related public resources of CAP database and its gene annotation. (**A**) The framework and linked public databases in CAP. (**B**) The major gene annotation page in CAP, including homologs, functional information, secondary structure and RNA-seq gene expression values in four tissues (callus, leaf, flower and fruit) and three mixtures of these tissues.

Precise gene prediction is one of the most important goals in genome annotation. We combined *ab initio* gene finding programs and evidence-based annotation including homology searches, EST, RNA-Seq and RNA-PET experimental evidence to identify protein-coding genes in sweet orange genome. Detailed process is described in [3]. In CAP website, gene annotation page provides convenient searching items for gene information. Users can search the system by gene locus, Gene Ontology (GO) [5], InterPro category [6] or functional information. Comprehensive gene annotation is linked to public resources (Figure 1A). Figure 1B illustrates the detailed gene annotation, including functional information in SwissProt [7], othologs in *Arabidopsis thaliana* and *Oryza sativa*, inparalogs in sweet orange, KEGG orthologs [4], GO [5] and Mapman [8] category, protein fingerprints in PRINTS [9], protein families and domains in Pfam [10] and SUPFAM [11], prediction RNA fold and protein secondary structure, as well as RNA-seq Reads Per Kilo bases per Million (RPKM) values for different sweet orange tissues. For the annotated protein-coding gene models, 93.5% have RNA-seq evidence support, 78.2% is supported with proteins in public non-redundant database, and 66.7% is supported with EST evidence, only a very small fraction of genes are solely predicted with *ab initio* gene-finding programs [3]. Table 1 lists the statistical information for functions of the protein-coding genes. More than 18,000 sweet orange genes have homologs in public databases. Furthermore, more than 26,000 protein-coding genes contain protein family and domain information. Only 4,930 genes have no functional information, which are annotated as “hypothetical proteins or conserved hypothetical proteins” (Table 1).

**Table 1:** Statistics of functional information for protein-coding genes in sweet orange.

Category	Description	Number
I	High similarity to known proteins in SwissProt (identity >90%) (identity >50%)intellectual (identity >90%)	524
II	Medium similarity to known proteins in SwissProt (identity >50%)	10,613
III	Low similarity to known proteins (identity >30%)	18,368
IV	InterPro domain-containing protein	26,916
V	Conserved hypothetical or hypothetical protein	4,930

### GBrowse

**Figure 2:**
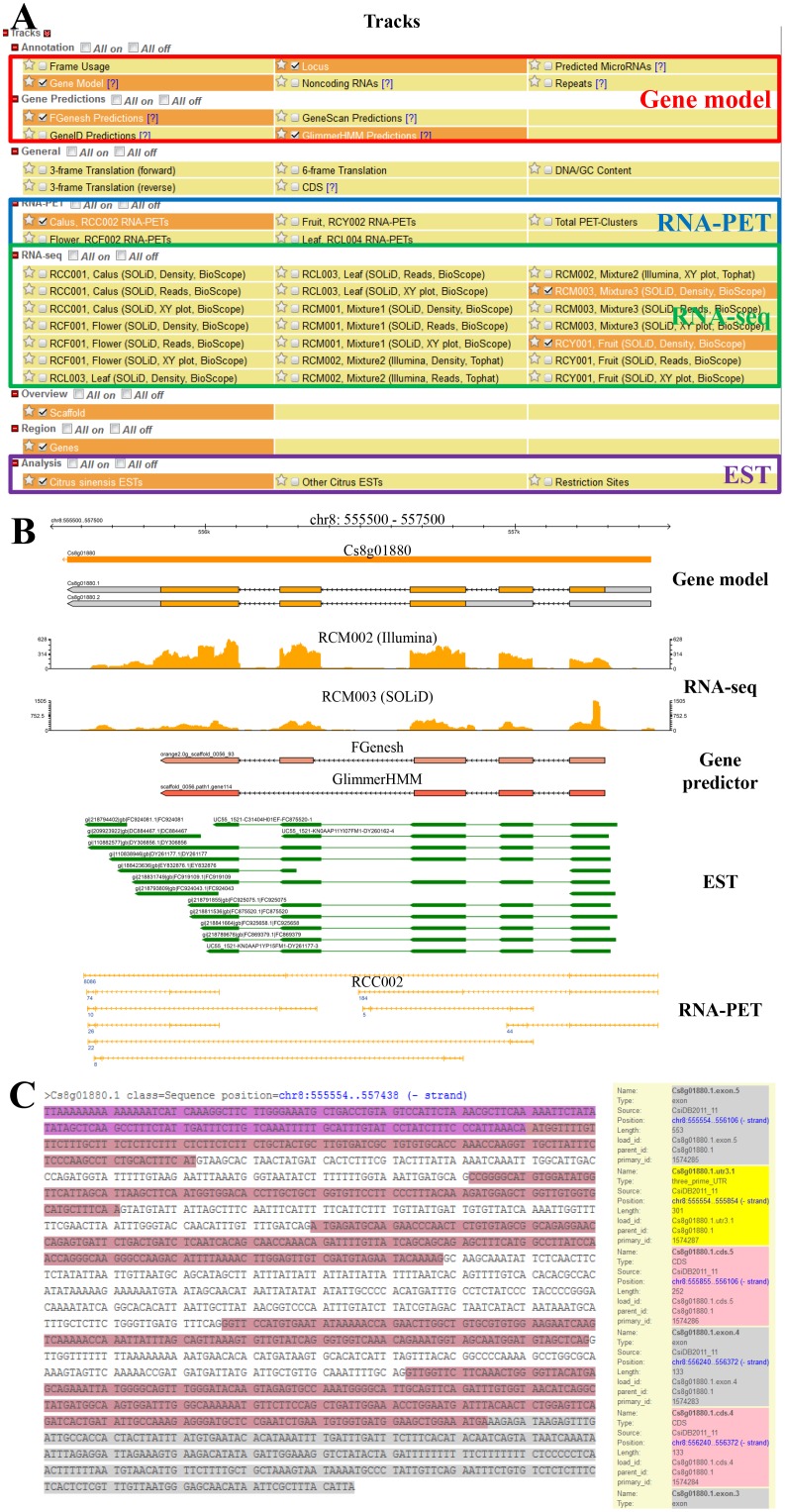
GBrowse in CAP. (**A**) GBrowse tracks in CAP. The tracks include general overview, gene model, RNA-seq, RNA-PET and EST evidence. (**B**) Graphic example of Cs8g1880 gene annotation in GBrowse. Gene model shows the exon-intron structure of the gene. Gene predictor shows the prediction results of some *ab initio* gene-finding programs. RNA-seq, RNA-PET and EST are the experimental evidence to support the gene model. (**C**) Text file of Cs8g1880 gene model in GBrowse.

It is well known that GBrowse is one of the most important genomic viewers for manipulating and displaying annotations on genomes [12], which has been extensively used to construct database for a variety of model organisms, such as Flybase [13], WormBase [14], SGD [15] and SilkDB [16]. CAP provides GBrowse-based integration of sweet orange genome annotation, including *ab initio* gene prediction, EST, RNA-seq and RNA-PET evidence-based gene annotation. Users can easily browse any interested regions in the sweet orange genome. According to the position on a scaffold, users can access a variety of track features, including scaffolds, protein-coding gene models, non-coding RNA, repetitive sequences, *ab initio* gene prediction, general information including GC content, 3-frame or 6-frame translation, RNA-seq and RNA-PET data from four sweet orange tissues (callus, leaf, flower and fruit) and three mixtures of these tissues, and ESTs from sweet orange and other citrus species (Figure 2A). Figure 2B illustrates a protein-coding gene Cs8g01880 in chromosome 8, detailed information includes the final gene model, RNA-seq and RNA-PET data from different tissues, ESTs from sweet orange and other citrus species, and four *ab initio* gene prediction tools, i.e., Genscan [17], GeneID [18], FgeneSH (http://linux1.softberry.com/berry.phtml?topic=fgenesh&group=programs&subgroup=gfind) and GlimmerHMM [19]. Gene mode page in GBrowse is available for each gene, including gene name, position, length, exon and intron position, 5′ and 3′ un-translated region, genomic sequence and transcripts (Figure 2C).

### Protein-protein interactions (PPIs)

**Figure 3:**
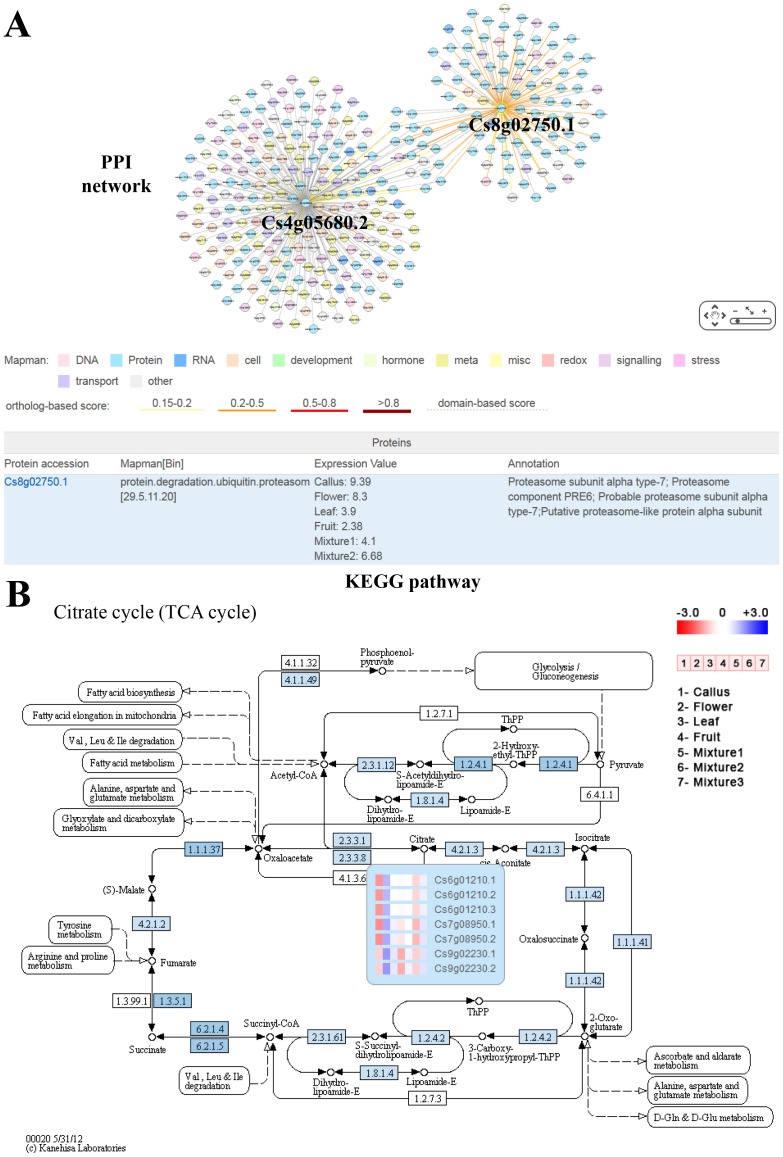
The predicted protein interactions and KEGG pathway in CAP. (**A**) The predicted PPI network of Cs8g02750.1 and Cs4g05680.2. (**B**) Citrate cycle (TCA cycle) metabolic pathway in sweet orange.

The sweet orange PPI network is predicted with ortholog-based and domain-combination methods, and then K-nearest neighbors (KNN) method is used to verify and filter the predicted PPIs, the final PPI network contains 124,491 interactions involving 8,195 proteins [20]. The web interface of PPI is constructed with JAVA and hosted on an Apache web server. The gene search page is linked to PPI, users can also submit one or more gene ID numbers to PPI search page, and then the server will return proteins that interact with the query proteins. The query protein and its interaction partners are visualized with Cytoscape software [21]. Figure 3A shows the PPI network of Cs8g02750.1 and Cs4g05680.2, Cs8g02750.1 is a proteasome subunit with 112 interacting partners, and Cs4g05680.2 is a serine/threonine-protein kinase with 236 interacting proteins. The two proteins have common and specific protein interactions. Cs8g02750.1 mainly interacts with other proteasome subunit proteins, while Cs4g05680.2 contains a wide variety of interacting partners, including many protein kinases, ribosomal proteins, 14-3-3 proteins, v-ATPases, tubulin proteins *etc*. In Figure 3A, nodes are colored according to Mapman functional categories [8]. Solid line between two nodes indicates interaction predicted with ortholog-based method, and dash line indicates interaction predicted with domain-combination method. Thickness and color of the solid line denotes different score levels, the higher the orthologous score, the thicker the line is. If a user clicks a node in the PPI network, its Mapman annotation, functional information and expression value will be shown (Figure 3A).

### Metabolic pathways

KEGG pathway maps are graphical diagrams representing knowledge of reaction networks for metabolism, and each map summarizes experimental evidence in published literatures [4]. Based on KEGG Orthology (KO) groups, we obtained the KEGG orthologous genes in sweet orange genome, and generated the sweet orange metabolic pathways. KEGG modules in each pathway map are produced by converting nodes to gene identifier nodes and colored in blue. Sweet orange pathways include four categories, *i.e.*, metabolism, genetic information processing, environmental information processing and cellular processes. Each category contains many pathways. When a user clicks a pathway, the reference KEGG pathway will be shown, and enzymes or proteins which have KEGG orthologs in sweet orange are colored in blue. Figure 3B shows the metabolic pathway of citrate cycle (TCA cycle). When mouse moves to an enzyme with blue color, orthologs in sweet orange and their expression values in different tissues are shown, which are also linked to the corresponding gene annotation.

### Search modules

CAP provides various query interface and graphical visualization to facilitate the retrieve and demonstration of sweet orange data. As mentioned above, “gene search” is the principal search system, which allows users to enter keywords such as gene locus, GO [5] or InterPro category [6], and functional information. The retrieving result links to GBrowse and PPI. Users can also submit the gene locus to search its protein interaction in the PPI menu. In addition, users can perform a BLAST sequence search to retrieve homologous sequences in sweet orange genome. BLAST search results include graphical summary of the sequence alignment, briefly and detailed description of the alignment. All the search results performed by the above search modules can be further used for functional investigation.

## Conclusions

The present work provides a comprehensive collection of sweet orange genomic and transcriptomic data, which are organized and deposited in an online database CAP. Convenient web interface is designed to show gene annotation, protein interaction and metabolic pathway. CAP serves the plant research community by providing a reference genome and annotation for sweet orange.

In the near future, CAP will collect the experimentally validated data for sweet orange genes. In addition, small RNA and degradome sequencing data will be added to CAP. New high-throughput DNA-sequencing technologies are being developed and it is expected that the number of *Citrus* species sequences will grow rapidly. These new sequences will be incorporated into the CAP by comparison to the *C. sinensis* reference genome in the future. With the update of sweet orange genome annotation, CAP will update to new version.

## Methods

### Data source and website architecture

The genomic data for sweet orange has been submitted to NCBI GenBank under the accession number AJPS00000000 and BioProject ID PRJNA86123. The raw data for sweet orange genome sequencing, assembling and annotation are available from sweet orange annotation project [3]. All the data are organized and stored in MySQL database (http://www.mysql.com/). Besides, the sequence information and functional annotation for protein-coding genes are provided in CAP. A genome browser is developed on the basis of GBrowse [12]. CAP is implemented in JSP language and deployed on Apache Tomcat web server (http://tomcat.apache.org/). The integrated network browser is created by Cytoscape web program (http://cytoscapeweb.cytoscape.org/) [21]. The architecture and linked public databases are shown in Figure 1A. CAP can be accessed through IE 6.0 or higher, Netscape 7.0 or higher, Safari, Opera, Chrome and Firefox from multiple platforms. JavaScript is required to use all the functions of CAP.

### Gene annotation and linked databases

SwissProt homologs are obtained by using BLASTP based on bi-directional best hit (BBH) method to search against UniProtKB/SwissProt [7]. Thresholds for BLASTP search are sequence coverage >0.7, identity >30%, e value <1e-10 and bit-score >60. Pfam category is predicted by using hmmer program [22]. Mapman annotation is obtained using BLASTP based on BBH method between *A. thaliana* and sweet orange genes. Gene3D, InterPro, PRINTS and SUPFAM annotation is predicted with Interproscan program [6]. RNA secondary structure is predicted with RNAfold program in ViennaRNA [23], and protein secondary structure is predicted with Psipred program [24].

Gene annotation in CAP is linked to many public databases. For example, Orthologs in *A. thaliana* and *O. sativa* are linked to gene model in TAIR [25] and MSU rice gene models (http://rice.plantbiology.msu.edu/), respectively. GO annotation links to gene ontology in EMBL database (http://www.embl.org/), Gene3D links to the corresponding CATH Superfamily (http://www.cathdb.info/), InterPro links to EMBL database (http://www.cathdb.info/), PRINTS links to SPRINT database (http://www.bioinf.manchester.ac.uk/dbbrowser/sprint/), Pfam links to corresponding Pfam category (http://pfam.sanger.ac.uk/), and SUPFAM links to superfamily database (http://supfam.org/).
